# Comparative Arthroscopic Rotator Cuff Repair Outcomes Associated with Continuous Interscalene Block vs. Bolus Interscalene Block with Intravenous Dexmedetomidine: A Retrospective Cohort Study

**DOI:** 10.3390/jcm14113882

**Published:** 2025-05-30

**Authors:** Seong-Meen Yoon, Aeryoung Lee, Sungwook Choi

**Affiliations:** 1 Department of Orthopedic Surgery, Jeju National University Hospital, Jeju National University College of Medicine, Jeju 63241, Republic of Korea; osyoon37182@gmail.com; 2 Department of Anesthesiology and Pain Medicine, Jeju National University Hospital, Jeju National University College of Medicine, Jeju 63241, Republic of Korea; nanrong95@naver.com

**Keywords:** interscalene block, postoperative pain, rotator cuff, shoulder arthroscopy

## Abstract

**Background/Objectives:** This retrospective cohort study compared the outcomes (postoperative pain, morphine consumption, sleep disturbance, and function) of patients undergoing arthroscopic rotator cuff repair involving either continuous interscalene block (CISB group) or single-injection interscalene block with intravenous dexmedetomidine (SISB group) analgesia. **Methods**: This study included 61 patients, aged 59–71 years, who underwent elective arthroscopic rotator cuff repair and for whom complete electronic health records were available. Patients in the SISB group received a single-injection nerve block plus intraoperative intravenous dexmedetomidine and postoperative patient-controlled analgesia (PCA; morphine and dexmedetomidine; *n* = 33). Patients in the CISB group received continuous nerve block and morphine PCA (*n* = 28). Patient sleep disturbances, pain, total morphine consumption, and functional outcomes were evaluated postoperatively. **Results**: During the first 36 h postoperatively, there were no significant differences in the measured effects for patients in the two groups. Similarly, there were no significant differences in functional outcomes. However, patients in the SISB group had a significantly lower median morphine consumption total (18 mg) than those in the CISB group (24 mg; *p* < 0.001). **Conclusions**: Patients in the SISB group demonstrated significantly lower median morphine consumption than those in the CISB group; however, the postoperative pain, frequency of sleep disturbances, nausea, and functional outcomes did not show statistically significant differences. The reduced morphine consumption associated with the SISB group, compared with the CISB group, suggests that this analgesic protocol may result in fewer opioid-related effects following arthroscopic rotator cuff repair.

## 1. Introduction

Currently, arthroscopic rotator cuff repair (ARCR) is the treatment of choice for rotator cuff tears [1], with approximately 90% of such cases being treated using ARCR in 2017 [2]. Rotator cuff pathology is often accompanied by other shoulder conditions, the most common of which is the long head of the biceps tendinopathy. These coexisting conditions can complicate both the surgical approach and postoperative rehabilitation [3]. Compared with open or mini-open procedures, ARCR is associated with significantly shorter admission periods and lower complication rates (e.g., fewer surgical site infections and less pain). Despite the better pain outcomes associated with arthroscopic surgery, many patients are unable to undergo early rehabilitation due to residual pain, ultimately delaying recovery times and increasing the rate of adhesions. Moreover, uncontrolled pain leads to patient dissatisfaction and poor sleep quality during hospitalization.

Following ARCR, interscalene/suprascapular blocks and axillary nerve blocks are recommended in addition to the use of dexamethasone, paracetamol, and nonsteroidal anti-inflammatory drugs for postoperative pain control [4]. Multiple studies have demonstrated that interscalene nerve blocks reduce the use of postoperative narcotic analgesics [5]. In addition, sufficient pain control decreases the bleeding rate during arthroscopic surgery, thereby improving the visual field during the operation [5,6]. Consequently, interscalene nerve blocks are now recommended for use in addition to general anesthesia. Options for their administration include an indwelling catheter with continuous infusion, single-bolus injection, and multiple blocks. Several recent reports have demonstrated the benefits of both an interscalene brachial plexus bolus blockade (IBPBB) and continuous infusion during ARCR.

Choi et al. [7] demonstrated that an IBPBB provides effective and immediate postoperative analgesia for up to 6 h. Salviz et al. [8] showed that mean visual analog scale (VAS) pain scores are lower for patients receiving continuous blocks than for patients receiving single blocks on postoperative days (PODs) 1 and 2, and narcotic usage was lower until POD 3. Abdallah et al. [9] demonstrated that a single block can provide effective analgesia for up to 8 h after surgery, whereas Fredrickson et al. [10] showed that continuous infusion reduced pain during the first two PODs. However, there is no definite guideline for the use of interscalene blocks. The use of perineural or intravenous (IV) additives, such as dexamethasone or dexmedetomidine, is known to prolong the duration of analgesia [11,12]. A study investigating the appropriate dose of dexmedetomidine reported that IV dexmedetomidine (2.0 μg/kg) significantly increases the time to first pain at the surgical site following an IBPBB [13,14].

This retrospective study was prompted by the perception that patients receiving a combination of a single-injection interscalene block with dexmedetomidine infusion (SISB) plus patient-controlled anesthesia (PCA) had improved patient outcomes compared with those receiving a continuous interscalene block (CISB) plus PCA. Thus, this study retrospectively examined patients undergoing ARCR involving these two pain control protocols with a hypothesis that there would be a difference in outcomes and/or in opioid consumption given the reported risks of opioid-related and CISB-associated side effects, including nausea and vomiting, motor weakness, neurologic deficits, and catheter-related issues such as infection or displacement [15,16,17].

## 2. Materials and Methods

### 2.1. Patient Selection

Patients who underwent elective unilateral arthroscopic shoulder surgery between May 2019 and March 2021 at Jeju National University Hospital were screened for eligibility in this study based on their electronic health records. Records were included for patients who met the initial screening requirements: 45–75 years old, positive shoulder examination test results, magnetic resonance imaging-based diagnosis of mid-sized rotator cuff tears, symptoms persisting for more than 3 months after conservative treatment, VAS score of greater than 5, American Society of Anesthesiologists physical status classification of I to III, and the same anesthesiologist performing the IBPBB. Patients received either CISB or SISB based on the attending anesthesiologist’s clinical judgment and equipment availability.

### 2.2. Intraoperative Management and Surgical Technique

Each patient received standardized general anesthesia with 3–6 mg/kg pentothal sodium and 0.8 mg/kg rocuronium, followed by tracheal intubation and maintenance with inhaled sevoflurane and IV remifentanil (0.01–0.1 μg/kg/min). Except for remifentanil, additional analgesics were not permitted intraoperatively. A continuous infusion of remifentanil was titrated to maintain the heart rate and/or mean blood pressure within 20% of the preinduction values. A decrease in the mean blood pressure of more than 20% of the baseline value was treated with 2.5–5 mg ephedrine; marked bradycardia (<50 beats/min) was treated with 0.25–0.5 mg atropine. Upon conclusion of the surgery, each patient was transferred to the post-anesthesia care unit and remained there until the criteria for discharge to the ward were met.

### 2.3. Interscalene Brachial Plexus Block Technique

The same anesthesiologist performed the IBPBB technique on each patient included in the study. Midazolam (1–2 mg, IV) was used for anxiolysis before the procedure, with supplemental oxygen provided via a nasal cannula. Following surgical site sterilization, the skin was infiltrated with 1 mL of 1% lidocaine. Subsequently, a sterile, 50 mm, 22-gauge, insulated nerve-stimulating needle (PAJUNK, Geisingen, Germany) was inserted, under ultrasonographic guidance, via the in-plane technique until the needle tip was adjacent to the C5 and C6 roots; the appropriate needle position was confirmed at a threshold current of less than 0.5 mA. After confirming the correct needle position using the negative blood aspiration test, 15 mL of 0.5% ropivacaine (epinephrine 1:200,000) was injected. After IBPBB, the sensory block was assessed every 5 min for 30 min using the pinprick test in the C5 and C6 sensory dermatomal distribution region. The extent of sensory loss was graded on a 3-point scale (2 = normal sensation, 1 = loss of sensation to pinprick, 0 = loss of sensation to light touch). Motor block was assessed using arm abduction (C5) and forearm flexion (C6) graded on a 3-point scale (2 = normal, 1 = weakness, 0 = complete loss of power). A successful nerve block was defined as a sensory score of 0 within 30 min following injection of the local anesthetic.

### 2.4. Continuous Interscalene Block Versus Single-Injection Interscalene Block with IV Dexmedetomidine

**Figure 1:**
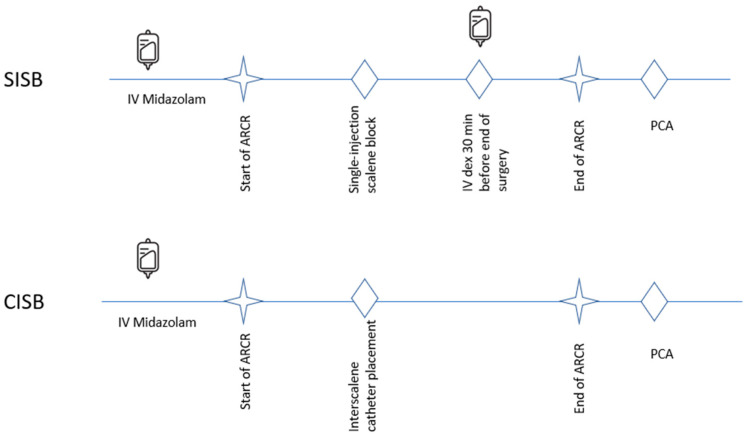
SISB vs. CISB scheme. ARCR, arthroscopic rotator cuff repair surgery; CISB, continuous interscalene block; dex, dexmedetomidine; IV, intravenous; min, minutes; PCA, patient-controlled analgesia; SISB, single-injection interscalene block. Notes: Following placement of the interscalene catheter in patients receiving CISB, continuous infusion of ropivacaine continued through to the end of the surgery. Patients undergoing SISB received only a single injection of ropivacaine. The PCA setup involved an on-demand 2 mL bolus of morphine with a lockout time of 8 min and a background infusion rate of 1 mL/h.

For patients receiving CISB, PCA was initiated using 0.5 mg/mL morphine in normal saline. For patients receiving SISB, an initial loading dose of 0.4 μg/kg dexmedetomidine was administered via IV infusion 0.5 h before the end of surgery, and PCA was initiated with 0.5 mg/mL morphine plus 1 μg/mL dexmedetomidine in normal saline (Figure 1). For all patients, the PCA was programmed to deliver an on-demand 2 mL bolus with a lockout time of 8 min and a background infusion rate of 1 mL/h. The cumulative PCA morphine consumption was determined at 2, 12, and 36 h postoperatively.

### 2.5. Patient Evaluations

Each patient was assessed for postoperative pain using the VAS score (postoperatively at 2, 12, and 36 h). For shoulder function, the University of California, Los Angeles (UCLA) shoulder score (0 represents the worst shoulder function, and 35 represents the best function) and the Constant score (0 [worst] to 100 [best]) were determined; the ranges-of-motion (ROMs) during forward flexion and abduction of the affected shoulder were also measured. These functional assessments of the shoulder were performed at admission and postoperatively at 3 and 6 months. Patients were also asked about the presence or absence of sleep disturbances on PODs 1 and 2 by answering questions regarding whether postoperative pain prevented them from going to sleep or caused them to be unable to stay asleep. Postoperative blood pressure values were also recorded for each patient; a systolic blood pressure value below 90 mmHg was considered to represent anesthesia-related hypotension.

### 2.6. Postoperative Management and Rehabilitation Protocol

All patients underwent a standardized postoperative rehabilitation protocol. For four weeks after the operation, a shoulder brace featuring 0° of external rotation and 30° of abduction was uniformly applied. Despite the relatively brief immobilization period following rotator cuff repair, exercises were initiated early, as the participants had medium-sized rotator cuff tears with minimal tendon retraction. Immediate allowances included shoulder shrugging and active movements of the fingers, wrist, and elbow. Pendulum exercises and gentle passive shoulder ROM activities were introduced on POD 3. Active-assisted shoulder ROM exercises were initiated following discontinuation of the brace, and resisted shoulder motion and strengthening exercises were introduced three months after surgery.

### 2.7. Statistical Analysis

All statistical analyses were performed using SPSS software for Windows (Version 17.0; SPSS, Chicago, IL, USA). Continuous variables were expressed as means ± standard deviations or medians and ranges, as appropriate. The chi-square test or Fisher exact test was used for categorical variables. Repeated-measures analysis of variance (ANOVA) was used to assess changes in functional outcomes over time within each group (preoperative, postoperative 3 months, and 6 months); the Tukey posthoc test was also applied. A *p* value < 0.05 was considered statistically significant. The statistical analysis was performed by the first author.

## 3. Results

**Figure 2:**
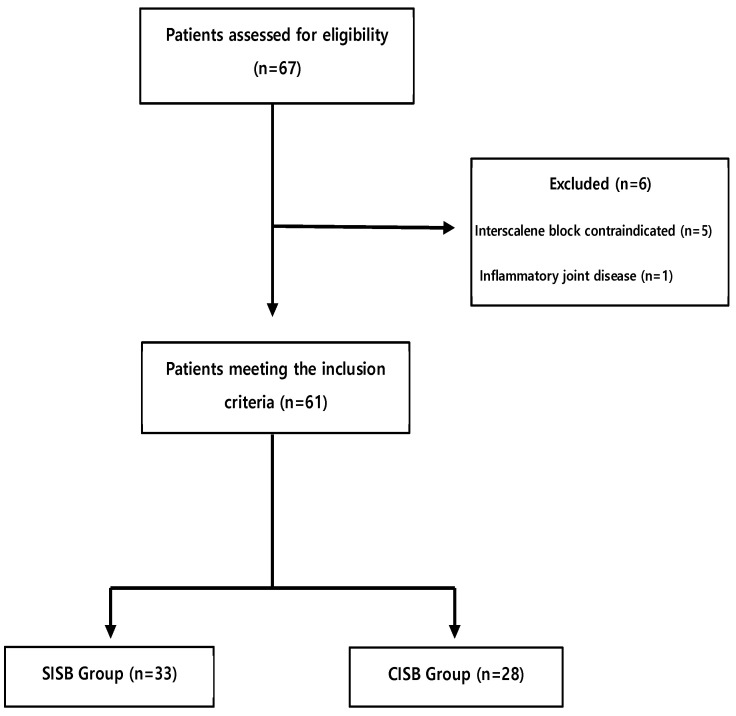
Flow diagram describing patient selection. CISB, continuous interscalene block; SISB, single-injection interscalene block.

This retrospective study was approved by the Jeju (Republic of South Korea) National University Hospital Ethics Board (identifier 2019-05-015); written informed consent was obtained from the patients included in the study. Of the 67 patient records meeting the initial screening requirements, 61 were eligible for inclusion in the study; 6 were excluded from the study for the reasons shown in Figure 2. All blocks and catheters were successfully placed, and all 61 patients (33 in the SISB group and 28 in the CISB group) were followed for at least six months after their surgery. The mean age (± SD) of the 61 patients was 64.5 ± 17 years (range, 59–71 years). The patient demographic data suggested that the SISB and CISB groups were similarly matched by sex and age (both, *p* > 0.5); moreover, there was no significant difference evident in the overall health of the patients, as assessed by the presence of chronic disease. All procedures were performed arthroscopically, and all patients underwent general anesthesia and interscalene block. The mean procedure length was 121 min, and the mean anesthesia time was 191 min. Pain relief and sleep disturbances were tracked during the first 36 h postoperatively.

**Figure 3:**
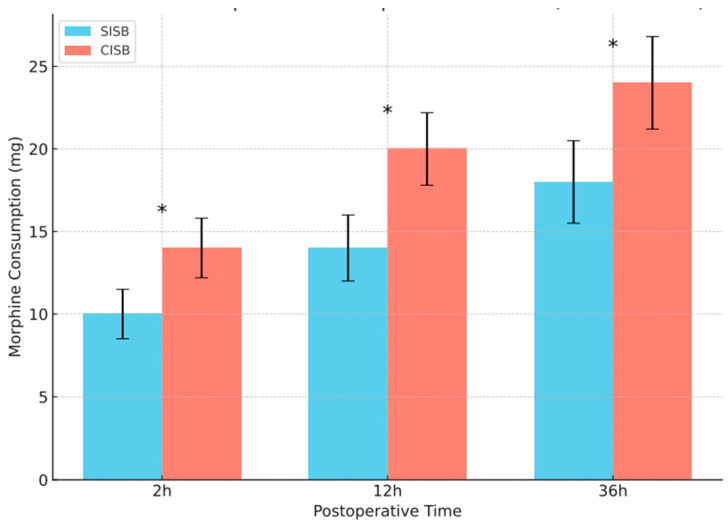
Cumulative total morphine consumption for patients in the SISB and CISB groups. CISB, continuous interscalene block; SISB, single-injection interscalene block. * *p* < 0.0001.

Patients in the SISB group had a significantly lower median total PCA morphine consumption at 36 h postoperatively (18 mg) compared with those in the CISB group (24 mg; Figure 3). The median morphine consumption totals for patients in the SISB group were also lower, compared with those in the CISB group, at 2 and 12 h after surgery (both, *p* < 0.001). Despite the reduced morphine consumption, the VAS scores for postoperative pain (Table 1) showed no significant differences between the two analgesic protocol groups at any time point.

There was a significant difference (*p* < 0.001) in the median total morphine consumption values at each time point.

In the SISB group, slightly more than half of the patients (57%) complained of sleep disturbances for two days after surgery, and four patients (12%) experienced anesthesia-related hypotension (Table 2). A tingling sensation persisted for one day in one patient (3.0%), and nausea developed in one patient (3.0%). In the CISB group, more than half of the patients (64%) complained of sleep disturbances for two days after surgery, and six patients (21%) showed anesthesia-related hypotension. Persistent sensory disturbances lasting for more than two days after completing the infusion occurred in three patients (10.7%), and nausea developed in three patients (10.7%) on POD 2. However, there were no remarkable complications in either group.

For the ROM evaluations, we assessed forward flexion and abduction preoperatively and at 3 and 6 months after arthroscopic shoulder surgery (Table 3). The average forward flexion of the affected shoulder increased by 8.2° in the SISB group and by 9.6° in the CISB group. Similarly, the average abduction of the affected shoulder increased by 17.6° in the SISB group and by 10.3° in the CISB group. Thus, the ROM evaluations indicated the patients in both groups experienced significant ROM improvements, compared with preoperative evaluations, but there were no between-group differences in the extent of improvement in forward flexion or abduction. The UCLA and Constant scores for each group also improved significantly, relative to preoperative values, by the final follow-up (both, *p* < 0.0001), but there were no significant differences in the mean functional scores between groups (Table 3).

## 4. Discussion

The current study showed that patients who underwent ARCR involving SISB and dexmedetomidine/morphine PCA did not experience statistically different amounts of postoperative pain or different numbers of anesthesia-related complications as those in the CISB group, but they did demonstrate a significantly lower median morphine consumption. Moreover, there were no significant differences in the functional outcomes for the patients in the two groups. The study results also suggest trends toward reduced postoperative pain (as indicated by the number of patients experiencing sleep disturbances), particularly on POD 1, and reduced nausea. Thus, this study suggests that SISB, combined with dexmedetomidine/morphine-based PCA, may provide improved pain control relative to CISB and does not result in significant differences in functional outcomes for the patients.

Previous studies investigating the effect of dexmedetomidine, alone, for IV PCA after gynecological laparoscopic operations have reported that dexmedetomidine decreases patient postoperative pain and significantly reduces the incidence of nausea and vomiting compared with patients receiving opioid-based pain control [17,18]. Li et al. [17] also showed that dexmedetomidine administration provided an antinausea effect by reducing morphine consumption while improving postoperative pain control. Their study reported that patients receiving dexmedetomidine consumed 29% less PCA morphine, and the incidence of nausea was significantly lower. In the present study, the cumulative consumption of morphine by patients in the SISB group was approximately 25% less than that consumed by patients in the CISB group. Although the incidences of postoperative pain, nausea, and vomiting were not significantly different between the groups in this study, the results suggest a trend toward fewer of these side effects with the reduced total morphine consumption. This outcome would be expected since opioids are the primary trigger of nausea and vomiting, and the opioid-sparing effect of dexmedetomidine would be expected to result in decreased nausea and vomiting.

Potent opioids result in hemodynamic changes after surgery. Several studies have investigated the effects of dexmedetomidine in patients undergoing various types of surgeries. Gerlach et al. [19] reported that dexmedetomidine administration for postoperative analgesia avoided the development of undesirable hemodynamic effects. Li et al. [20] compared patients who underwent abdominal total hysterectomies with postoperative pain relief involving IV PCA morphine alone and in combination with dexmedetomidine. They did not observe cases of bradycardia, hypotension, oversedation, or respiratory depression, suggesting that the addition of dexmedetomidine to IV PCA resulted in superior analgesia and a significant morphine-sparing effect. In the present study, the use of a dexmedetomidine/morphine PCA protocol resulted in 33% fewer patients (not a statistically significant difference) in the SISB group experiencing postoperative anesthesia-related hypotension compared with those receiving only PCA morphine. This may have been the result of the combined effects of the sympathoinhibitory effects of dexmedetomidine and the direct peripheral dilation from morphine.

Continuous peripheral nerve blocks appear to have a higher likelihood of neurological sequelae due to the prolonged infusion of local anesthetics and the long indwelling period of the perineural catheter [21]. In patients who received CISB, Borgeat et al. [22] reported minor neurological complications at 1, 3, and 6 months postoperatively. In the present study, three patients in the SISB group experienced a tingling sensation for 1 day and one patient experienced sensory disturbance. In the CISB group, three patients experienced a tingling sensation for 2 days. All patients completely recovered from their neurological deficits except one patient in the CISB group who complained of residual sensory weakness for 3 weeks after the surgery. According to previous studies, transient motor and sensory disturbances are often encountered after CISB; however, complete neurological deficits are rare [23]. These neurological complications cause patients to be quite uncomfortable and anxious, which might reduce the level of patient satisfaction concerning postoperative pain control.

Furthermore, catheter dislodgement or migration, variable infusion rates, bleeding, and breakage have been reported in patients receiving CISB [15]. Other studies (e.g., Byeon et al. [24] and Lee et al. [25]) have reported that catheter retention is problematic in patients receiving CISB. This is generally caused by shoulder mobilization exercises when the patient starts to rehabilitate (1 day after rotator cuff repair surgery). Moreover, the patients are beginning to engage in a wider range of postoperative activities than previously due to their improved ROM. Thus, CISB catheter retention around the neck and shoulder has the potential to be problematic.

### Limitations

There are several limitations to this study. The retrospective nature of this study limits the conclusions that may be drawn from the study for a variety of reasons, including the inability to assure the randomization of patient assignments to particular groups and the inability to fully blind the investigators to patient assignments. Although the patient demographics indicated that there were no significant differences in the age, sex, and overall health of the participants in each group, the nature of the study prevented any conclusions regarding the presence of confounding variables that could have affected the clinical and functional outcomes. The limited size of our patient population may have prevented the observation of significant differences in some outcomes (postoperative pain and nausea). In addition, we did not consider the effect of nonsteroidal anti-inflammatory drugs in controlling postsurgical pain after ARCR, as these were not tracked in the electronic health records. The study’s focus on patients with medium-sized rotator cuff tears enhanced the homogeneity of our sample, thereby strengthening the internal validity of our comparisons. However, this also limits the generalizability of our findings to other tear sizes. Future studies should explore whether similar outcomes are observed in patients with small, large, or massive rotator cuff tears, as postoperative pain and recovery trajectories may differ.

## 5. Conclusions

In this retrospective cohort study, we compared two postoperative pain control strategies in patients undergoing ARCR. Our findings indicate that SISB with intravenous dexmedetomidine resulted in significantly lower morphine consumption while not resulting in statistically significant differences in pain relief, sleep quality, or functional outcomes compared with CISB. Given the reduced opioid use and similar efficacy, SISB with dexmedetomidine may be considered a viable alternative for postoperative analgesia. Future prospective studies with larger samples are recommended to validate these results.

## Figures and Tables

**Table 1:** Serial changes in postoperative pain scores.

Postoperative Time (h)	Visual Analog Scale Score		
	SISB Group (*n* = 33)	CISB Group (*n* = 28)	*p* Value
2	1.6 ± 5.0	2.7 ± 4.5	0.716
12	5.8 ± 2.5	6.2 ± 2.1	0.618
36	4.5 ± 2.5	4.5 ± 2.2	0.940

CISB, Continuous interscalene block; SISB, single-injection interscalene block. All values represent mean ± standard deviation.

**Table 2:** Sleep quality and anesthesia-related complications.

	SISB Group (*n* = 33)	CISB Group (*n* = 28)	*p* Value
Sleep disturbance due to pain			
POD 1	14 (42.4%)	18 (64.3%)	0.148
POD 2	19 (57.6%)	18 (64.3%)	0.786
Anesthesia-related hypotension	4 (12.1%)	6 (21.4%)	0.490
Sensory disturbance			
POD 1	1 (3.0%)	3 (10.7%)	0.434
POD 2	0 (0.0%)	3 (10.7%)	0.715
Nausea or vomiting			
POD 1	1 (3.0%)	3 (10.7%)	0.241
POD 2	0 (0.0%)	3 (10.7%)	0.226

CISB, continuous interscalene block; POD, postoperative day; SISB, single-injection interscalene block.

**Table 3:** Serial changes in functional outcomes.

	Preoperative		3 Months Postoperative		6 Months Postoperative	
Evaluation	SISB Group (*n* = 33)	CISB Group (*n* = 28)	SISB Group (*n* = 33)	CISB Group (*n* = 28)	SISB Group (*n* = 33)	CISB Group (*n* = 28)
UCLA	18.4 ± 4.5	19.1 ± 3.9	26.4 ± 5.1 <0.0001 ^a^	25.2 ± 4.1 <0.0001 ^a^	30.3 ± 3.5 <0.0001 ^b^ 0.00030 ^c^	31.3 ± 3.8 <0.0001 ^b^ 0.0001 ^c^
Constant	43.3 ± 15.0	40.5 ± 15.2	79.3 ± 14.4 <0.0001 ^a^	74.3 ± 12.2 <0.0001 ^a^	87.6 ± 9.7 <0.0001 ^b^ 0.1042 ^c^	88.3 ± 10.2 <0.0001 ^b^ 0.0011 ^c^
ROM: FF (degrees)	170.0 ± 7.5	167.5 ± 5.9	175.7 ± 5.3 0.0009 ^a^	173.4 ± 4.9 0.0019 ^a^	178.2 ± 4.5 <0.0001 ^b^ 0.4745	177.1 ± 5.3 <0.0001 ^b^ 0.1480
ROM: ABD (degrees)	160.0 ± 6.4	165.0 ± 6.4	170.2 ± 5.8 <0.0001 ^a^	169.8 ± 5.1 0.0254 ^a^	177.6 ± 5.8 <0.0001 ^a^ <0.0001 ^b^	175.3 ± 4.7 <0.0001 ^a^ 0.0059 ^b^

CISB, continuous interscalene block; ABD, abduction; FF, forward flexion; ROM, range of motion; SISB, single-injection interscalene block; UCLA, University of California, Los Angeles. Notes: Values are shown as means ± standard deviation. The UCLA and Constant scores are dimensionless. There was no statistical difference between the SISB and CISB groups for any evaluation at any time point (*p* values ranged from 0.5234 to 0.9999). The statistical analysis was performed using analysis of variance, followed by the Tukey post-hoc test. ^a^ *p* value comparing the 3-month postoperative evaluation with the preoperative evaluation. ^b^ *p* value comparing the 6-month postoperative evaluation with the preoperative evaluation. ^c^ *p* value comparing the 6-month postoperative evaluation with the 3-month postoperative evaluation.

## Data Availability

The raw data supporting the conclusions of this article will be made available by the authors upon reasonable request.

## Abbreviations

The following abbreviations are used in this manuscript:

ARCR	Arthroscopic rotator cuff repair
CISB	Continuous interscalene block
IBPBB	Interscalene brachial plexus bolus blockade
IV	Intravenous
PCA	Patient-controlled analgesia
POD	Postoperative day
ROM	Range of motion
SISB	Single-injection interscalene block with intravenous dexmedetomidine
UCLA	University of California, Los Angeles
VAS	Visual analog scale
